# *Clostridium butyricum* MIYAIRI 588 contributes to the maintenance of intestinal microbiota diversity early after haematopoietic cell transplantation

**DOI:** 10.1038/s41409-024-02250-1

**Published:** 2024-03-02

**Authors:** Kentaro Fukushima, Hayami Kudo, Kentaro Oka, Atsushi Hayashi, Makoto Onizuka, Shinsuke Kusakabe, Akihisa Hino, Motomichi Takahashi, Kiyoshi Takeda, Masaki Mori, Kiyoshi Ando, Naoki Hosen

**Affiliations:** 1 Department of Haematology and Oncology, Osaka University Graduate School of Medicine, Suita, 565-0871 Japan; 2 R&D Division, Central Research Institute, Miyarisan Pharmaceutical Co., Ltd., Saitama, 331-0804 Japan; 3 Department of Hematology/Oncology, Tokai University School of Medicine, Isehara, 259-1193 Japan; 4 Laboratory of Immune Regulation, Department of Microbiology and Immunology, Graduate School of Medicine, Osaka University, Suita, 565-0871 Japan; 5 World Premier International Immunology Frontier Research Centre, Osaka University, Suita, 565-0871 Japan; 6 Integrated Frontier Research for Medical Science Division, Institute for Open and Transdisciplinary Research Initiatives (OTRI), Osaka University, Suita, 565-0871 Japan; 7 Faculty of Medicine, Tokai University School of Medicine, Isehara, 259-1193 Japan

**Keywords:** Risk factors, Translational research

## Abstract

In patients undergoing haematopoietic stem-cell transplantation (HSCT), the intestinal microbiota plays an important role in prognosis, transplant outcome, and complications such as graft-versus-host disease (GVHD). Our prior research revealed that patients undergoing HSCT substantially differed from healthy controls. In this retrospective study, we showed that administering *Clostridium butyricum* MIYAIRI 588 (CBM588) as a live biotherapeutic agent is associated with maintaining intestinal microbiota in the early post-HSCT period. Alpha diversity, which reflects species richness, declined considerably in patients who did not receive CBM588, whereas it remained consistent in those who received CBM588. In addition, β-diversity analysis revealed that CBM588 did not alter the gut microbiota structure at 7–21 days post-HSCT. Patients who developed GVHD showed structural changes in their microbiota from the pre-transplant period, which was noticeable on day 14 before developing GVHD. *Enterococcus* was significantly prevalent in patients with GVHD after HSCT, and the population of *Bacteroides* was maintained from the pre-HSCT period through to the post-HSCT period. Patients who received CBM588 exhibited a contrasting trend, with lower relative abundances of both genera *Enterococcus* and *Bacteroides*. These results suggest that preoperative treatment with CBM588 could potentially be beneficial in maintaining intestinal microbiota balance.

## Introduction

The intestinal microbiota plays a crucial role in maintaining human health by regulating digestion, metabolism, and immune function [1–3]. Haematopoietic stem cell transplantation (HSCT) is a curative therapy for various haematological malignancies and non-malignant disorders [4–6]. Innovations in amplicon sequencing technology, conditioning chemotherapy, HSCT, and subsequent antimicrobial treatment have been shown to significantly influence intestinal microbiota [7–10]. Time-dependent analyses of the intestinal microbiota before and after transplantation have shown that the stability of the intestinal microbiota positively influences prognosis not only during the peri-transplant period but also over the long term [11–13].

Recently, living organisms with clear effects on diseases have been designated as live biotherapeutic products (LBPs), a new classification that differs from conventional probiotics [14–16]. *Clostridium butyricum* MIYAIRI 588 (CBM588), a spore-forming anaerobic bacterium, is recognised and categorised as an LBP [17], and is used for the treatment and prevention of diarrhoea or constipation symptoms related to microbial dysbiosis in some countries, particularly in Japan [18]. CBM588 also exhibits tolerance to various stress factors, such as low pH and antimicrobials; therefore, CBM588 is effective in treating and preventing antibiotic-associated diarrhoea in children by normalising the intestinal microbiota disturbed by antibiotics [19]. Administering CBM588 in mice improved the imbalance of microbial conditions caused by antibiotics [20]. Specifically, CBM588 increased *Bifidobacterium*, *Lactobacillus*, and *Lactococcus* abundance in the gut. Likewise, similar results were observed that *Bifidobacterium spp*. increased in patients with renal cell carcinoma who responded to CBM588 with immune-checkpoint inhibitor treatment [21]. Microbial treatment using probiotics, prebiotics, and faecal microbiota transplantation are associated with therapeutic efficacy in patients with HSCT [22–26]. However, the efficacy of microbial intervention approaches after HSCT is debatable, owing to a lack of knowledge regarding their safety and mechanism of action [27]. A randomised pilot study (NCT03922035) is currently underway to assess the safety and feasibility of CBM588 administration during the peri-transplant period and to compare the incidence and severity of adverse events [28].

In this study, we aimed to evaluate the effects of prophylactic CBM588 administration on the intestinal microbiota after HSCT by using 16 S rRNA amplicon sequencing.

## Materials and methods

### Patients

This study was approved by the sites’ institutional review boards and conducted in accordance with the Declaration of Helsinki. All patients provided written informed consent. Forty patients who underwent allogeneic HSCT at Osaka or Tokai University Hospital between May 2006 and May 2017 were included. All enroled patients were of Japanese ethnicity. Data collected for analysis included age at transplantation, sex, conditioning regimen, date and cause of death, and the incidence and severity of acute graft-versus-host disease (aGVHD). The definitions of myeloablative conditioning or reduced-intensity conditioning regimens were based on previous methodology, with slight modifications [29, 30]. The reliability of GVHD diagnosis in all patients was assessed using confidence levels defined by The Mount Sinai Acute GVHD International Consortium consortium [31]. CBM588, known as Miya-BM (Miyarisan Pharmaceutical Co., Ltd., Tokyo, Japan), is an approved medication for improving digestive symptoms and is commonly used in Japan. Patients were administered 60 mg of oral CBM588 daily in addition to the usual postoperative antimicrobials.

### Collection of faecal samples

Baseline faecal samples were collected 7 days before HSCT, i.e., before initiation of HSCT conditioning regimens. Subsequently, we sequentially obtained one sample per week from the initiation of the conditioning regimen until 35 days after HSCT. Patients placed the faecal samples into disposable sterile faeces tubes (76 × 20 mm; Sarstedt, Nümbrecht, Germany), and the samples were preserved in RNAlater™ stabilisation solution (Thermo Fisher Scientific, Waltham, MA, USA) at −80 °C. Faecal samples were collected from all 40 patients. Patients whose samples were not accurately collected every 7 days were excluded. Patients were also excluded if their samples were unavailable within a two-day window either before or after each designated time point, namely −7, 0, 7, 14, 21, 28, and 35 days. Faecal samples (196) from a sub-cohort of 37 patients were analysed via this procedure.

#### DNA extraction

Bacterial DNA was extracted as described previously using a technique involving mechanical cell lysis with bead-beating regimens [32]. The extracted DNA was purified using the High Pure Polymerase Chain Reaction (PCR) Template Preparation Kit (Roche Diagnostics) following the manufacturer’s instructions.

### 16S rRNA gene sequencing and data processing

PCR targeting the V3–V4 region of the bacterial 16 S rRNA gene was performed using Ex Taq Hot Start (Takara Bio Inc.) for 35 cycles. The specific universal primer pairs 341 F (5′-CCTACGGGNGGCWGCAG) and 805 R (5′-GACTACHVGGGTATCTAATCC) were used in this study. PCR products were purified using SPRISElect beads (Beckman-Coulter Inc., California, USA). The amplified DNA was quantified using the ONEdsDNA System (Promega, Madison, WI, USA) and a Quantus fluorometer (Promega). The PCR amplicon libraries were prepared by pooling approximately equal amounts of amplified DNA and sequencing on an Illumina MiSeq platform (Illumina, San Diego, USA) using a 2 × 300 v3 600-cycle kit (Illumina) following the manufacturer’s instructions.

### Data processing

Illumina MiSeq fastq raw reads were analysed using the QIIME 2 platform (version 2019.10) [33]. The sequences were demultiplexed using the DADA 2 programme [34]. Amplicon sequence variants obtained using DADA 2, which were more accurate than the traditional operational classification units (OTUs), were assigned through feature-classifier classify-sklearn and SILVA database (version 138.1) [35]. Diversity analysis was performed using the QIIME diversity core metrics phylogenetic method [32].

### Quantitative PCR

Absolute quantification using quantitative PCR (qPCR) was performed to investigate whether the species used in LBPs (*C. butyricum*) increased in the intestinal tract [36]. The following primer sets were utilised: total bacteria, 5′-CGGYCCAGACTCCTACGGG-3′, and 5′-TTACCGCGGCTGCTGGCAC-3′; and *C. butyricum*, 5′- AGTGATTGTCAGTAGTAGACGAGCG-3′ and 5′-CATGCGCCCTTTGTAGC-3′. The assay was conducted on a Thermal Cycler Dice Real-Time System II (Takara Bio, Shiga, Japan) using TB Green® Premix Ex Taq™ II (Takara Bio).

### Statistical analysis

All statistical analyses were performed using the R software (version 4.1.1). Differences in alpha diversity and qPCR copies between groups were determined using the Mann–Whitney *U* test without any correction. For comparing numerical values of diversity distances, the Mann–Whitney *U* test and Welch’s *t*-test were used. Community composition was visualised using principal coordinate analysis (PCoA). PCoA was conducted on a comprehensive distance matrix encompassing the entire sample set. The significance of the groups in the community structure was tested using a permutational multivariate analysis of variance (PERMANOVA) with 9999 permutations. Spearman’s rank correlation coefficient was used to analyse the correlation for the relative abundance of bacterial genera against the first two principal coordinates (PC), namely PC1 and PC2. To estimate unique microbial genera in both groups treated with CBM588 and those without CBM588, DESeq2 was utilised. This tool assumes that read counts follow a negative binomial distribution [37].

## Results

### Patient cohort

**Fig. 1:**
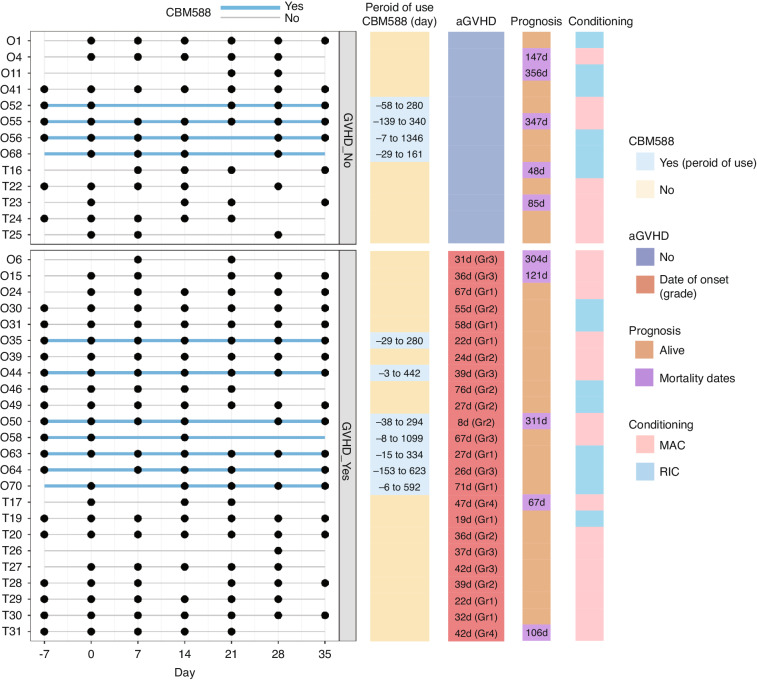
A selected sub-cohort of patients who underwent HSCT at two university hospitals was evaluated. The black circles represent observations taken over time. Periods of CBM588 administration are highlighted with blue lines. The right side of the chart presents additional data, including the onset of aGVHD, its severity grading, post-HSCT mortality dates (De), and the details of the preparatory conditioning regimen, MAC or (RIC). aGVHD Acute graft-versus-host disease, HSCT Haematopoietic stem cell transplantation, CBM588 *Clostridium butyricum* MIYAIRI 588, MAC Myeloablative, RIC Reduced-intensity conditioning.

The characteristics of the patient cohort are summarised in Fig. 1 and Supplementary Tables 1 and 2. The mean age of the 37 patients during HSCT was 44 years (range: 20–64 years). The study population comprised 15 females and 22 males; 13 patients (35.1%) experienced grade 0 aGvHD, whereas 24 patients (64.9%) developed mild-to-severe aGVHD (grades 1–4), with a median onset of 37 days post-transplantation. All patients received antibiotics before and after transplantation. Levofloxacin was administered orally to all patients as a prophylactic antimicrobial. During the neutropenic phase, cefepime, a fourth-generation cephalosporin, was administered to 15 patients (40.5%). Additionally, carbapenems, including biapenem, meropenem, or imipenem, were administered to 33 patients (89.2%). CBM588 was administered only to patients at Osaka University Hospital; therefore, 11 patients (29.7%) received CBM588, and 26 patients (70.3%) did not. The 1-year survival rates of patients who received preventive CBM588 administration and those who did not were 81.8% and 69.2%, respectively. The survival rate did not differ significantly between the groups.

### Dissimilarity of the bacterial community from that observed pre-HSCT

**Fig. 2:**
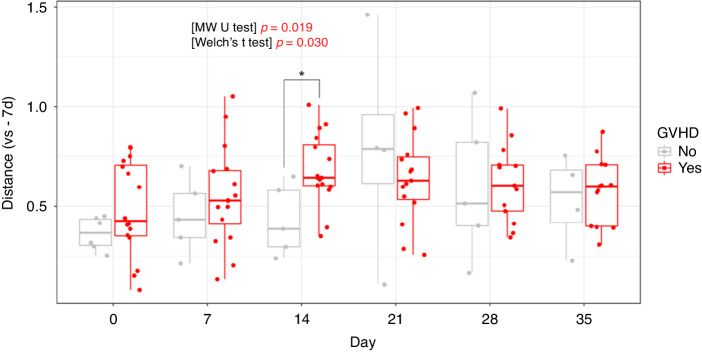
Boxplots depicting the variation in the weighted UniFrac distance between the pre- and post-HSCT microbiome at each time point, where Day -7 represents the pre-HSCT state. The asterisks indicate significant differences in distance values between patients who developed aGVHD post-HSCT and those who did not. These differences were assessed using the Mann–Whitney *U* test (*p* ≤ 0.05). HSCT Haematopoietic stem cell transplantation, aGVHD Acute graft-versus-host disease.

We demonstrated the fluctuations in community changes pre-HSCT and for each respective time point using the weighted UniFrac distance of beta diversity (Fig. 2). The greater the alterations in the microbiota, the more pronounced the distance. The temporal microbiome distance between the pre-HSCT and post-HSCT exhibited demonstrable augmentation from day 14 to day 21. A comparison between patients who developed aGVHD and those who did not reveal a discernible modification in the microbiota of patients with aGVHD at 14 days post-HSCT compared with that before HSCT (mean = 0.68; range in patients with aGVHD, 0.35–1.01).

Additionally, we investigated community changes with and without lower gastrointestinal GVHD development, which contributes a great deal to the loss of diversity, and with and without CBM588 administration, but no significant differences were detected (Fisher’s exact test: *p* = 0.443; chi-square test: *p* = 0.474) in this cohort (Supplementary Fig. 1).

### Association of the microbiota with aGVHD after aHSCT

**Fig. 3:**
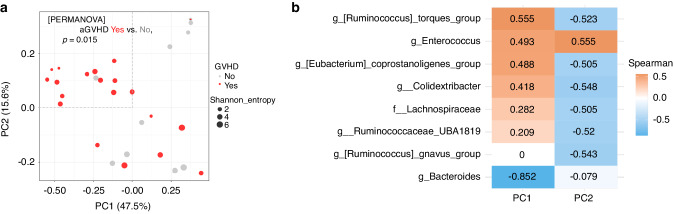
Structural changes in the intestinal microbiota and abundance of major bacterial groups in GVHD cases. **a** PCoA using weighted UniFrac distances highlighting the differences in the microbiome community structure at day 14 between individuals who developed aGVHD of grade I or higher and those who did not. **b** Heatmap showing the *r* values for the relative abundance of bacterial genera plotted against the first two principal coordinate values (PC1 and PC2). PCoA Principal coordinate analysis, aGVHD Acute graft-versus-host disease, *r* values, Correlation coefficients.

The microbiota of patients who developed aGVHD were compared with those who did not. The two groups showed no significant differences in alpha diversity at any of the sampling time points (data not shown). Conversely, PCoA based on the weighted UniFrac distance revealed significantly different taxonomic structures at 14 days post-HSCT between patients with aGVHD and those without aGVHD (Fig. 3a and Supplementary Fig. 2). Notably, the PCoA plots for patients who developed aGVHD were concentrated below 0.0 for PC1 and 0.0 for PC2. A correlation analysis of bacterial abundance with PC1 and PC2 was performed (Fig. 3b). The bacterial taxonomic structure of patients who developed aGVHD was characterised by a strong negative correlation with PC1 for the genus *Bacteroides* (*r* = −0.852) and a weak positive correlation with PC2 for the genus *Enterococcus* (*r* = 0.555).

### Impact of CBM588 consumption on the gut microbiota in allo-HSCT recipients

**Fig. 4:**
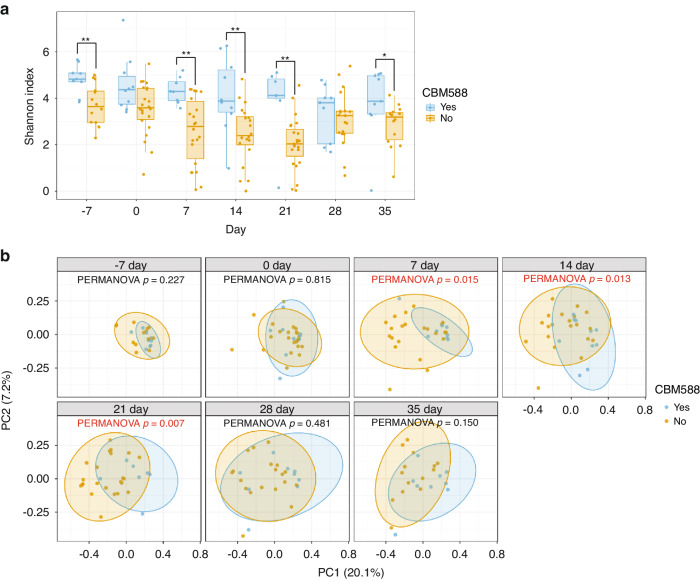
*Clostridium butyricum* MIYAIRI 588 administration preserves faecal microbiota diversity in the early post-transplant period. **a** Figure representing the alpha diversity of the faecal microbiota in the following two groups during the early post-transplant period: the CBM588 and no-administration groups (Control). Asterisks highlight significant differences in the Shannon indices of the microbiome between two groups, as determined using the Mann–Whitney *U* test (*p ≤ 0.05, **p ≤ 0.01). **b** PCoA based on the unweighted UniFrac distance of the microbiome over time. Statistical calculations were performed using the Adonis test and involved 999 permutations. PcoA Principal coordinate analysis, CBM588 *Clostridium butyricum* MIYAIRI 588.

HSCT conditioning regimens are known to alter the gut microbiota [38]. Initially, we tracked the variations in alpha diversity from baseline (before the conditioning regimen) to day 35 between patients who received CBM588 and those who did not (Fig. 4a). As anticipated, the median alpha diversity of patients who did not receive CBM588 declined gradually from 3.64 before transplantation to 2.03 after transplantation, with the peak decline observed on day 21. Conversely, among patients who received CBM588, the median alpha diversity remained consistent from baseline to day 21, ranging from 4.82 to 4.12. A two-dimensional representation of the unweighted UniFrac distance matrix was constructed using PCoA (Fig. 4b). A statistically significant divergence in the classification structure from 7 to 21 days was observed between the group that received CBM588 and the group that did not (PERMANOVA; *p* < 0.05). Moreover, 16 S metagenomic analysis provides reliable identification at the phylum to genus level but shows limited resolution at the species level [39]. Therefore, using qPCR, which is highly quantitative at the bacterial species level, we confirmed that *C. butyricum* outgrowth, the primary component, occurred only in the CBM588 treatment group (Supplementary Fig. 3).

### Temporal changes in the dominant bacteria with CBM588 administration

**Fig. 5:**
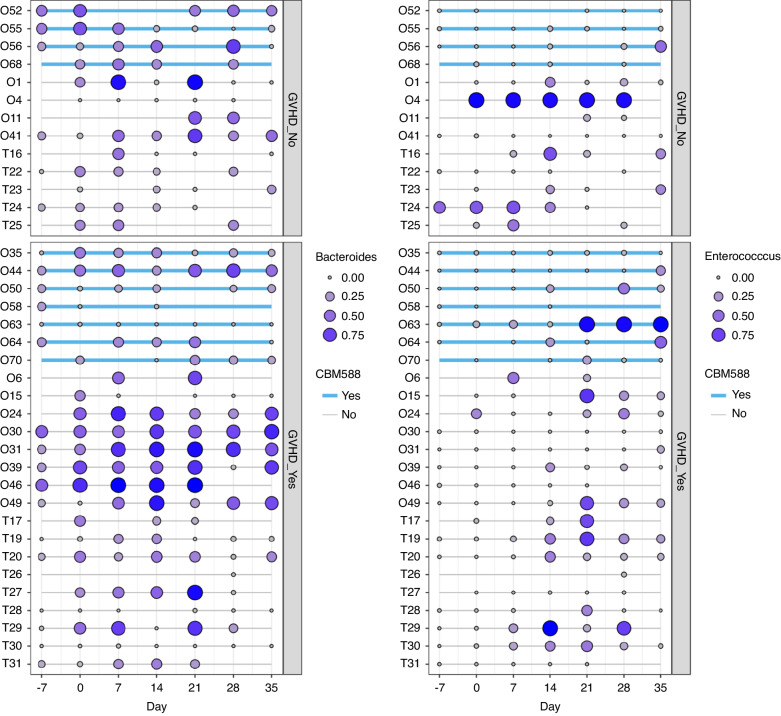
Swimmer plots illustrate the relative abundance of two bacterial genera, *Bacteroides* (left) and *Enterococcus* (right), in individual patients at each time point. The size of the circles corresponds to the relative abundance of each genus. The presence or absence of GVHD is differentiated by the upper and lower boxes, respectively, whereas the presence or absence of CBM588 is indicated by a blue line. CBM588 *Clostridium butyricum* MIYAIRI 588, GVHD Graft-versus-host disease.

Since *Bacteroides* and *Enterococcus* were abundant on day 14, the temporal variations in the abundance of these two bacterial genera were investigated (Fig. 5). The results indicated that the genus *Enterococcus* was more abundant in the microbiota of patients who developed aGVHD after day 14, whereas the genus *Bacteroides* was more prevalent among patients with aGVHD not only on day 14 but throughout the entire period from pre-HSCT to post-HSCT. The swimmer plot illustrates on a patient-by-patient basis whether CBM588 was administered or not (indicated by the blue line). Remarkably, patients who received CBM588 for prophylaxis exhibited a lower relative abundance of genus *Bacteroides* throughout the observed period. For instance, patients with O63 demonstrated low levels of genus *Bacteroides* at all observed periods, even if the patient had developed GVHD.

To capture the dominated community state type, all samples were subjected to cluster analysis, ignoring the time series. The Ward linkage hierarchical clustering method, which utilises a weighted UniFrac metric, was used. According to the segmented phylogenetic tree, four clusters were generated (Supplementary Fig. 4). Cluster 1 was characterised by the phylum Firmicutes, with a preponderance of the genus *Enterococcus*; Cluster 2 was characterised by the phylum Firmicutes, with the prevalence of the genera *Blautia* and *Rumicococcus*; Cluster 3 was typified by the phylum Bacteroidetes, dominated by the genus *Bacteroides*; and Cluster 4 exhibited a mixed microbiome consisting of both phyla Firmicutes and Bacteroidetes. We focused on days 7, 14, and 21 when post-transplant variability was high (Supplementary Fig. 5). According to the results of our cluster analysis, Cluster 4 consistently dominated the group that received CBM588, with proportions at each time point being 50.0%, 60.0%, and 42.9%, respectively. In contrast, in the group that did not receive CBM588, Cluster 3, represented by Bacteroides, accounted for 27.3%, 30.0%, and 43.5%, respectively.

We used DESeq2 to identify significantly different ASVs. Consistent with the cluster analysis results, we observed a significant elevation in genus *Enterococcus* levels in patients who did not receive CBM588 during the 7-day post-HSCT period (*p* < 0.05 and log2 fold change = 5.55). Additionally, patients who did not receive CBM588 exhibited higher levels of the genus *Bacteroides* on day 21 post-transplant (*p* < 0.05 and log2 fold change = 3.59).

## Discussion

Numerous studies have examined the link between gut microbiota and post-HSCT outcomes. Our initial report highlights the potential of using a single bacterium, CBM588, to intervene and modulate the gut microbiota after HSCT.

Previous studies have reported an association between dysbiosis and acute GVHD [40, 41]. As reported by Kusakabe et al., the sequential analysis of changes in the gut microbiota over time revealed that patients with large fluctuations in their microbiota had a poor prognosis [12]. To prove this, we compared the robustness of the microbiota of patients who developed aGVHD with that of patients who did not develop aGVHD by comparing the beta diversity distance matrix between the pre-HSCT and post-HSCT samples. Our results showed that at 14 days post-HSCT, patients who developed aGVHD had greater microbiota dissimilarity from pre-HSCT than those without aGVHD, suggesting that intervention and stabilisation of the gut microbiota before and early post-HSCT could help protect against GVHD.

In the early post-aHSCT period, patients who developed aGVHD exhibited lower microbial diversity [42–44]. These studies indicate a heightened risk of aGVHD-related complications associated with reduced bacterial diversity. Our study, based on the weighted UniFrac distance, revealed that patients who developed aGVHD had a significantly different microbiota composition compared to those who did not at 14 days post-HSCT. Therefore, changes in the composition of the gut microbiota may affect the host’s immune response and contribute to GVHD development. The combination of a correlation analysis of the composition of PCoA and microbiota with a focus on bacteria showed that the abundance of the genera *Bacteroides* and *Enterococcus* characterised the microbiota of the aGVHD group. Consistent with our findings, *Enterococcus* is reportedly associated with worsened GVHD [43, 45]. However, some studies have reported that the genus *Bacteroides* is abundant in non-GVHD cases [8], and others have reported expansion of the genus *Bacteroides* in studies of children with HSCT recipients and GVHD mouse models [46, 47]. Differences in temporal dynamics, recipient age, and host species may be responsible for these discrepancies in results among the studies. This heterogeneity can be resolved by higher-resolution species-level analyses.

In patients undergoing HSCT, the appropriate use of prophylactic antibiotics to control the intestinal environment while limiting the development of bacterial infections of intestinal origin is complicated [48]. Consequently, these patients tend to show reduced diversity of intestinal bacteria, resulting in a less diverse microbial community in comparison with the pre-transplant levels [49, 50]. In our study, the intestinal microenvironments of patients without CBM588 treatment consistently showed decreased bacterial diversity. In contrast, the alpha diversity of the patients receiving CBM588 treatment remained unchanged from the initial baseline, and the beta diversity of their overall bacterial structure also showed no significant changes. Oral administration of the probiotic *Lactobacillus plantarum* is safe and feasible in children and adolescents undergoing HSCT [51]. However, the genus *Lactobacillus* is sensitive to quinolone antibiotics, which are widely used for intestinal sterilisation, making *Lactobacillus* reaching the intestine difficult when quinolones are administered adequately. In contrast, CBM588, a spore-forming bacterium, maintains high biostability in the gut, suggesting that CBM588 does not alter the overall gut microbiota, which may be disrupted following transplantation. Additionally, alpha diversity was compared between the two universities to address potential confounding factors. Significant differences were observed only 7 days before transplantation, with no notable differences afterwards. This indicates that variations in facilities do not significantly affect the microbiota post-transplantation.

This study demonstrated that the abundance of genera *Enterococcus* and *Bacteroides* contributes to GVHD development. Throughout the observation period, their consistent abundance was confirmed. The abundance of the genus *Bacteroides* was lower in patients who received CBM588 than in those who did not. Cluster analysis on days 7, 14, and 21 shortly after transplantation also revealed a noticeably lower proportion of *Bacteroides* and *Enterococcus* in the CBM588-treated group. The gut microbiota of patients with haematological cancers is considerably different from that of healthy individuals [11, 12]. From these findings, by stabilising the microbiota, CBM588 could prevent the predominance of bacteria potentially associated with post-transplant prognosis.

A limitation of this study is that the sample size was not large enough to identify significant differences in the prognosis and outcomes with CBM588 administration. The small sample limited our ability to detect a significant difference between patients with and without lower gastrointestinal acute GVHD, as the onset of lower gastrointestinal GVHD occurred at or after day 21. Moreover, the evaluations on days 14 and 21 might have been preferentially influenced by the broad-spectrum antibiotics that almost all patients were using than by the lower gastrointestinal GVHD that they were about to develop. Thus, further prospective multicentre studies are needed to better characterise the variables affecting these changes and address the influence of microbiota-based therapeutics on allo-HSCT recipients. Moreover, given that some studies suggest that gastrointestinal GVHD is challenging to diagnose solely based on clinical symptoms, [52, 53] a comprehensive approach, incorporating endoscopic and histological evaluations with microbiota assessment, should be employed.

In conclusion, our data support the findings of previous reports showing that variations in gut bacteria influence clinical outcomes, including GVHD development. Furthermore, this is the first study to show that the administration of a microbiota-based therapeutic agent, CBM588, minimises post-transplant microbial fluctuations. Collectively, our findings uncover longitudinal microbiota maintained using CBM588, an LBP, and provide new insights for future therapeutic strategies.

### Supplementary information

Supplemental material

Table S1

Table S2

## Data Availability

The datasets generated during and/or analysed during the current study are available from the corresponding author on reasonable request.
