# Supplementary material for: Clostridium butyricum MIYAIRI 588 contributes to the maintenance of intestinal microbiota diversity early after haematopoietic cell transplantation
Source: Bone Marrow Transplant. 2024 Mar 2;59(6):795–802. doi: 10.1038/s41409-024-02250-1 (PMC11161410; doi:10.1038/s41409-024-02250-1)
Supplement: Supplementary file 1 — Supplemental material [file 41409_2024_2250_MOESM1_ESM.pdf]

Supplementary Figure 1 |

Trajectories of beta diversity with and without lower GI aGVHD before, at the time of, and after HSCT.
Changes in the composition of the microbiota between patients with and without GVHD do not show significant differences post-HSCT.

aGVHD, Acute graft-versus-host disease; GVHD, Graft-versus-host disease; HSCT, Haematopoietic stem cell transplantation

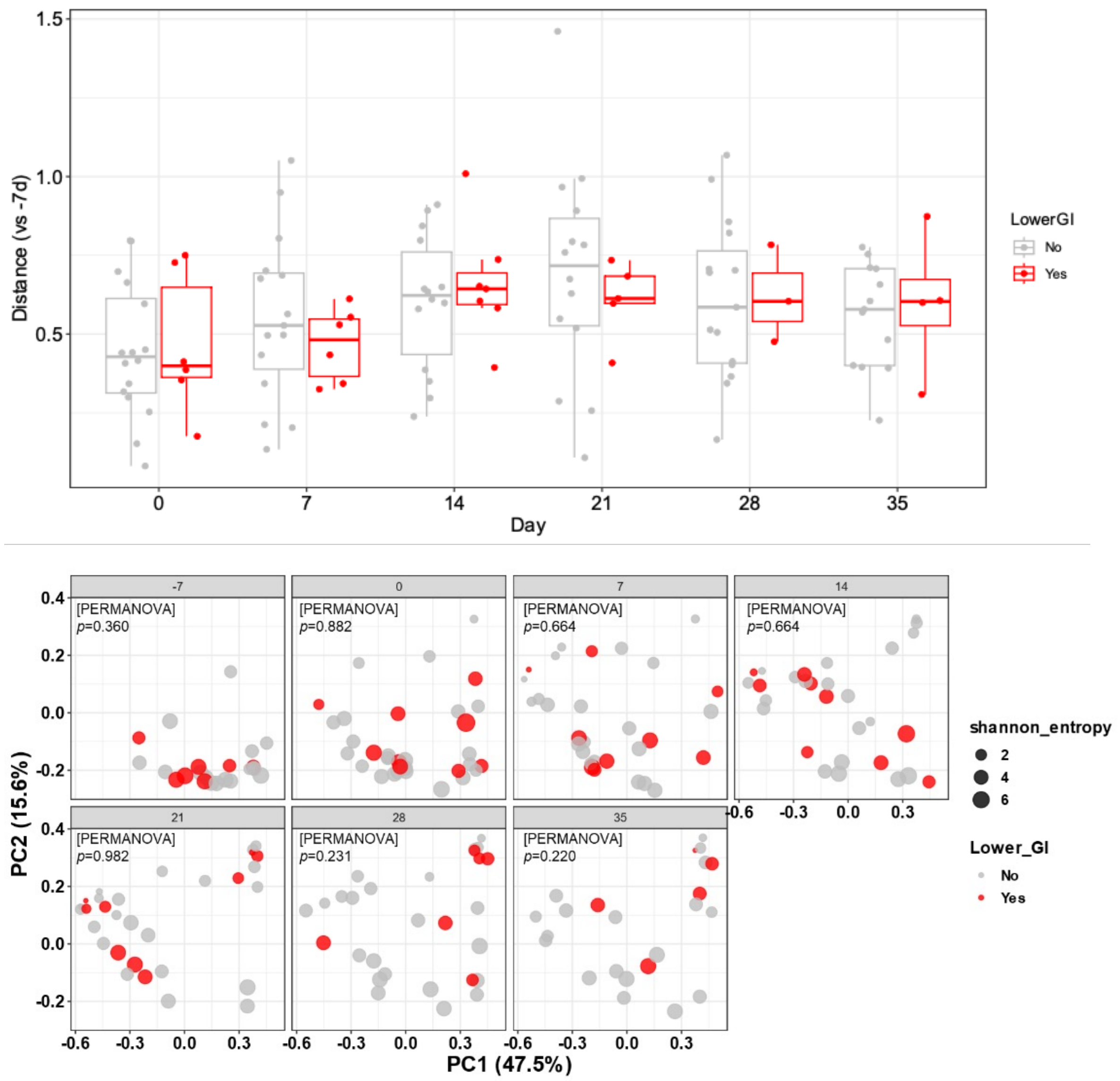

Supplementary Figure 2 | Trajectories of beta diversity in relation to aGVHD, before, at the time of, and after HSCT.Changes in the composition of the microbiota between patients with and without GVHD show a marked difference only on day 14 post-HSCT.
aGVHD, Acute graft-versus-host disease; GVHD, Graft-versus-host disease; HSCT, Haematopoietic stem cell transplantation

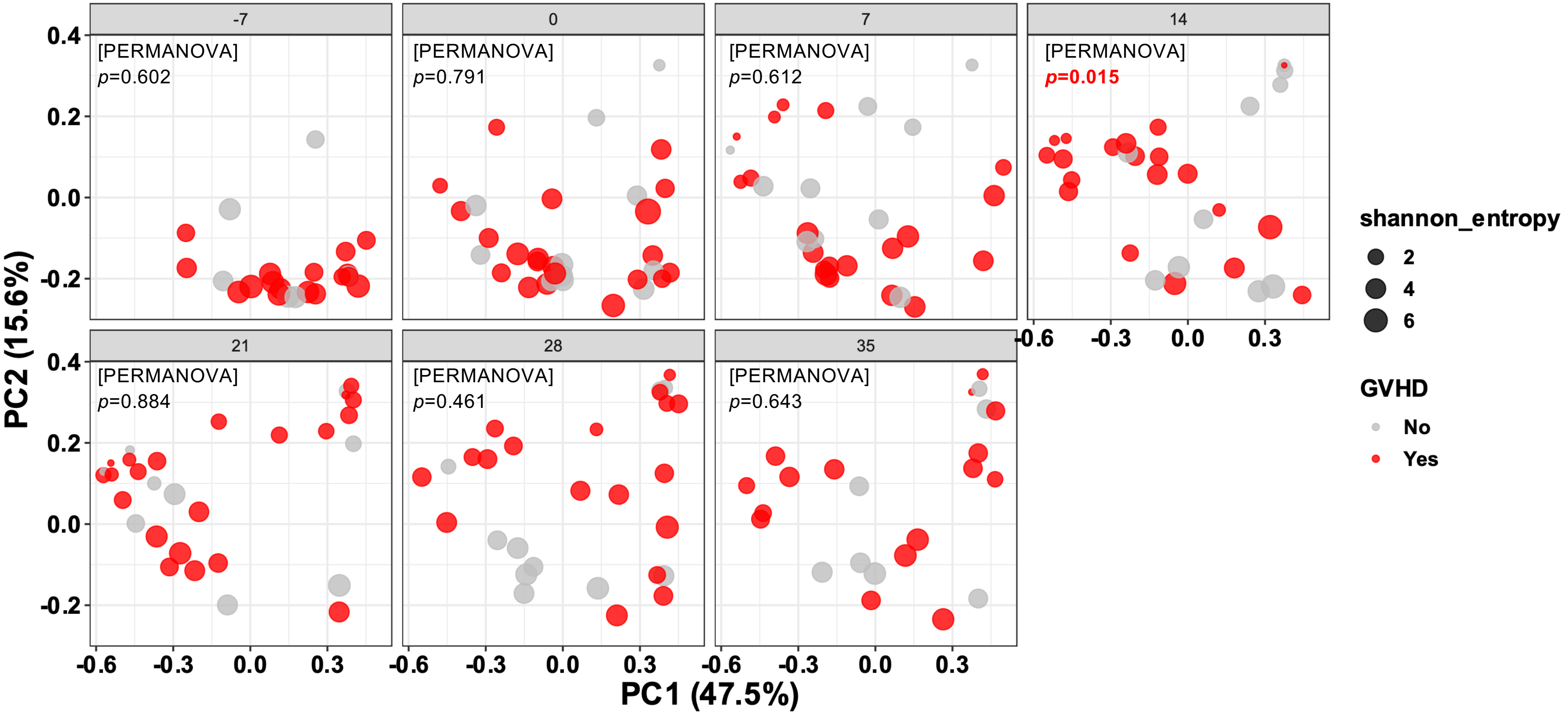

Supplementary Figure 3 | *C. buryricum* was detected in the administration group for the entire study period

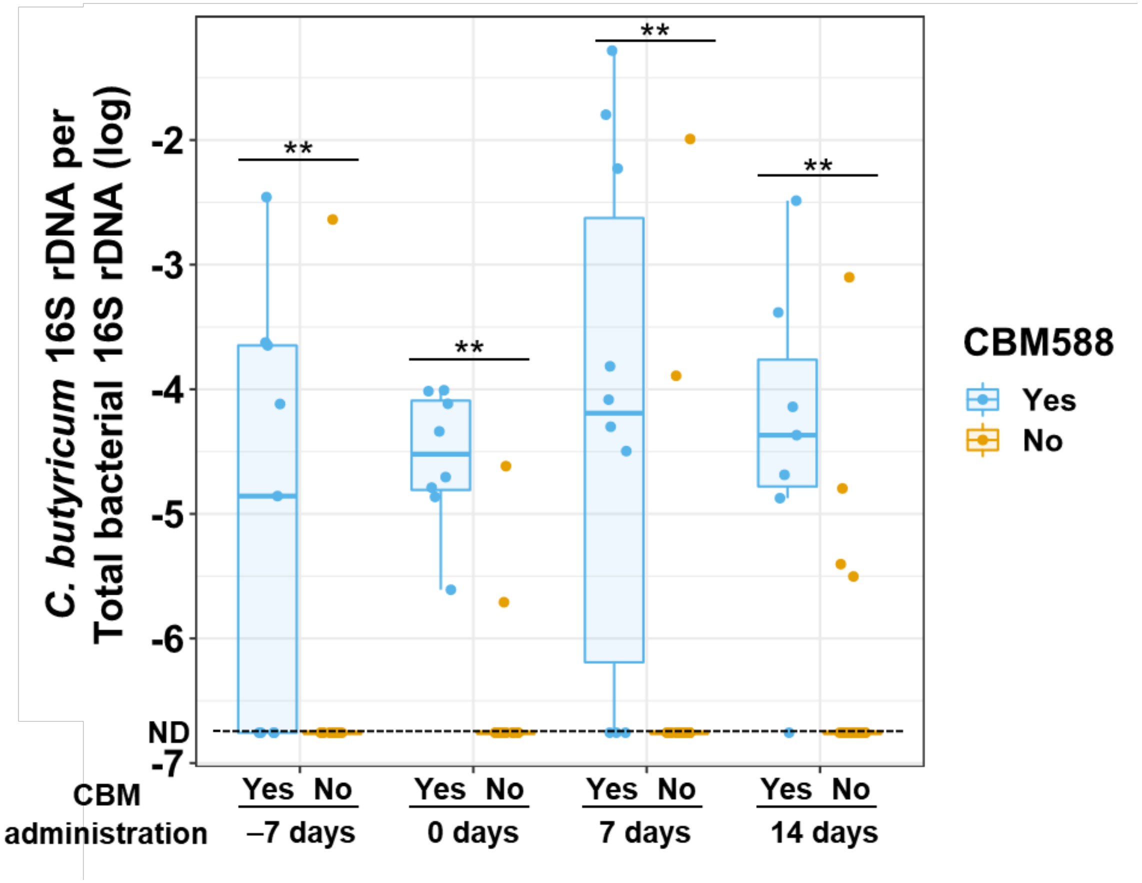

Since 16S rRNA metagenomic analysis provides resolution only up to the genus level, we examined the faecal abundance of *C. butyricum* using species-specific quantitative PCR (qPCR). The boxplot depicts the qPCR analysis of *C. butyricum* normalised to the total bacterial counts. Notably, *C. butyricum* was consistently detected in patients who received prophylactic CBM588 throughout the study period.

qPCR, Quantitative polymerase chain reaction; CBM588, Clostridium butyricum MIYAIRI 588

Supplementary Figure 4 | Clustering on microbiota distance-based analysis.

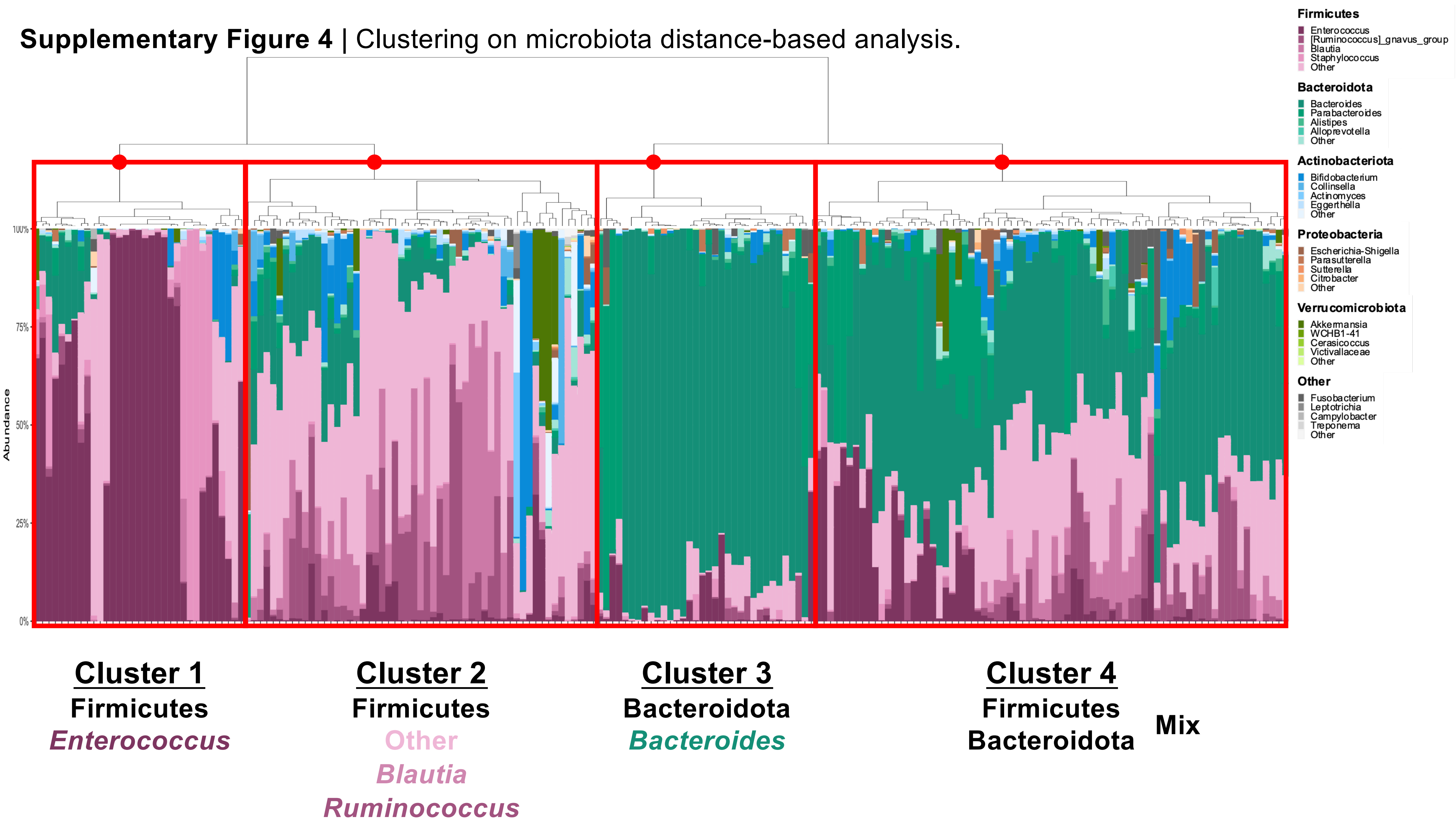

Supplementary Figure 4 | Clustering on microbiota distance-based analysis

Ward’s linkage hierarchical clustering analysis was performed using the weighted UniFrac metric. The analysis included all microbiota data derived from samples taken at every time point without considering patient data or clinical parameters such as CBM588 administration or the manifestation of GVHD. The horizontal axis represents the individual data points, whereas the vertical axis represents the distance or similarity between the points. The lengths of the branches reflect the distance and similarity between clusters, and the junctions of these branches denote the point at which a new cluster is formed. This process resulted in the approximate delineation into four unique clusters.

GVHD, Graft-versus-host disease; CBM588, Clostridium butyricum MIYAIRI 588

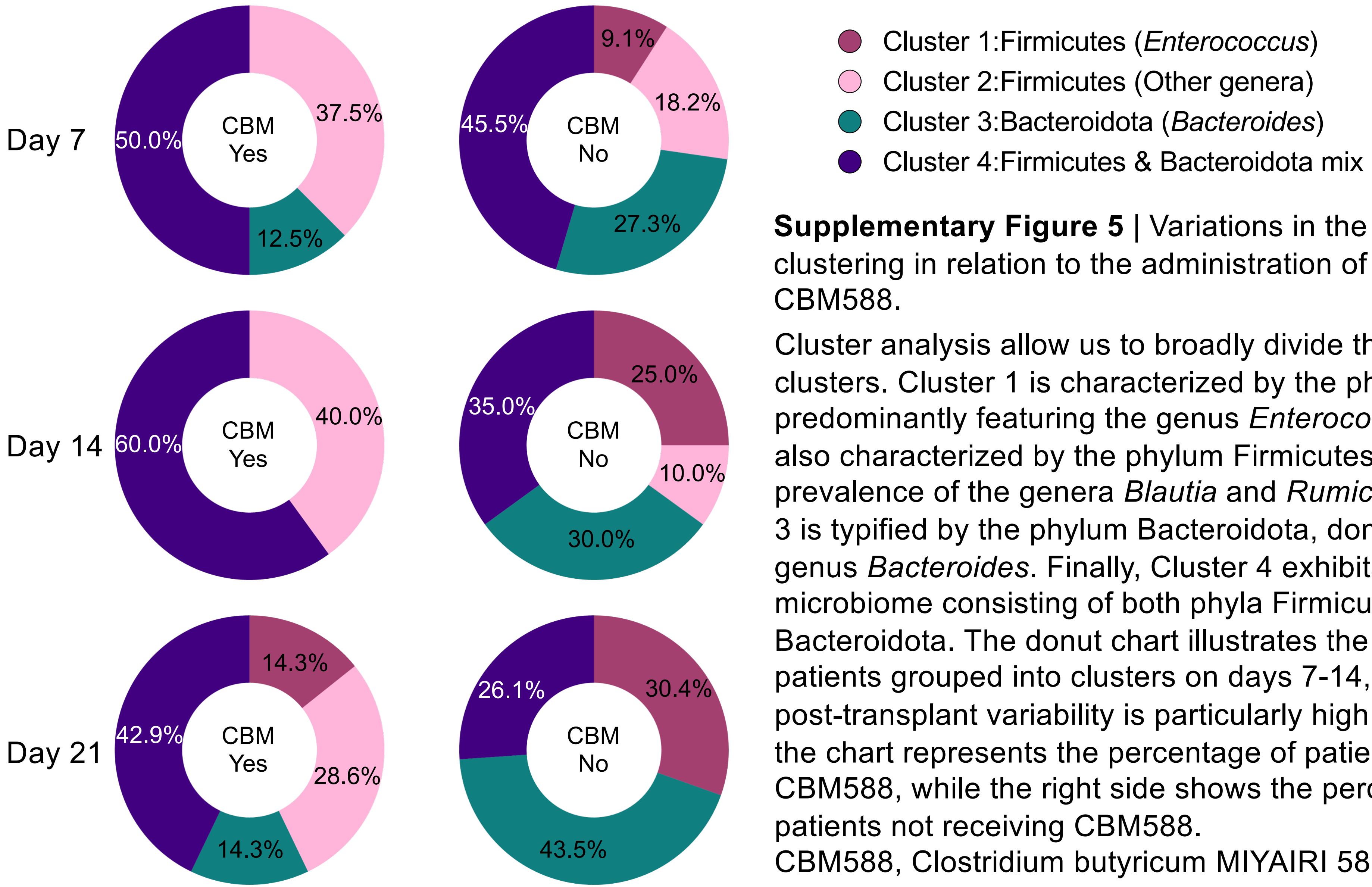

Supplementary Figure 5 | Variations in the microbiota clustering in relation to the administration of prophylactic CBM588.

Cluster analysis allow us to broadly divide the data into four clusters. Cluster 1 is characterized by the phylum Firmicutes, predominantly featuring the genus *Enterococcus*. Cluster 2, also characterized by the phylum Firmicutes, shows a prevalence of the genera *Blautia* and *Rumicococcus*. Cluster 3 is typified by the phylum Bacteroidota, dominated by the genus *Bacteroides*. Finally, Cluster 4 exhibits a mixed microbiome consisting of both phyla Firmicutes and Bacteroidota. The donut chart illustrates the proportion of patients grouped into clusters on days 7-14, a period when post-transplant variability is particularly high. The left side of the chart represents the percentage of patients receiving CBM588, while the right side shows the percentage of patients not receiving CBM588.
CBM588, Clostridium butyricum MIYAIRI 588

Table S1. Clinical patient characteristics

Characteristic		Number of patients (%)			Number of fecal samples (%)		
		Total	Osaka University	Tokai University	Total	Osaka University	Tokai University
Transplant recipients		37	23 (62.2)	14 (37.8)	–	–	–
Average recipient age (range)		44 (20-64)	46 (24-64)	40 (20-58)			
Patient sex	Female	15	7 (46.7)	8 (53.3)	–	–	–
	Male	22	16 (72.7)	6 (27.3)	–	–	–
Administration of CBM588	Yes	11	11	0	–	–	–
	No	26	12	14	–	–	–
Time Points	Before	–	–	–	23	15 (65.2)	8 (34.8)
	HSCT	–	–	–	32	20 (62.5)	12 (37.5)
	At 7 days	–	–	–	30	19 (63.3)	11 (36.7)
	At 14 days	–	–	–	30	19 (63.3)	11 (36.7)
	At 21 days	–	–	–	30	19 (63.3)	11 (36.7)
	At 28 days	–	–	–	28	19 (67.9)	9 (32.1)
	At 35 days	–	–	–	23	17 (73.9)	6 (26.1)
Acute GVHD	Yes	24	15 (62.5)	9 (37.5)	–	–	–
	No	13	8 (61.5)	5 (38.5)	–	–	–
Conditioning regimen	MAC	23	11 (47.8)	12 (52.2)	–	–	–
	RIC	14	12 (85.7)	2 (14.3)	–	–	–
Overall survival*	Alive	27	17 (63.0)	10 (27.0)	–	–	–
	Dead	10	6 (60.0)	4 (40.0)	–	–	–

CBM588 ; Clostridium butyricum MIYAIRI588, *Before* ; before conditioning, *MAC* ; myeloablative conditioning, *RIC* ; reduced intensity conditioning,
* indicates overall survival rate at one year

Table S2. Detailed Patients Characteristics.

No	PatientID	University	Age	Sex	Disease	Relation	Source	CBM	Conditioning	_clinical_gr	D_days	SR_1yrs	Skin	gut	Liver	LVFX	CFPM	AMK	Carbapenems
1	O_1	Osaka	56	Male	LBL	Unrelated	BMT	No	RIC	Zero	849	Alive	0	0	0	Yes	No	No	Yes
2	O_4	Osaka	33	Male	ALL	Related	PBSCT	No	MAC	Zero	147	Dead	0	0	0	Yes	No	No	Yes
3	O_11	Osaka	27	Male	Other	Related	PBSCT	No	RIC	Zero	356	Dead	0	0	0	Yes	No	No	Yes
4	O_41	Osaka	52	Female	AML	Unrelated	BMT	No	RIC	Zero	490	Alive	0	0	0	Yes	No	No	Yes
5	O_52	Osaka	37	Female	AML	Related	BMT	Yes	MAC	Zero	531	Alive	0	0	0	Yes	No	No	Yes
6	O_55	Osaka	38	Male	ALL	Unrelated	BMT	Yes	MAC	Zero	347	Dead	0	0	0	Yes	No	No	Yes
7	O_56	Osaka	51	Male	AML	Unrelated	BMT	Yes	RIC	Zero	1029	Alive	0	0	0	Yes	No	No	Yes
8	O_68	Osaka	63	Female	AML	Unrelated	BMT	Yes	RIC	Zero	587	Alive	0	0	0	Yes	No	No	No
9	T_16	Tokai	51	Female	ATL	Unrelated	CBSCT	No	RIC	Zero	48	Dead	0	0	0	Yes	No	No	Yes
10	T_22	Tokai	32	Female	AML	Related	BMT	No	MAC	Zero	3534	Alive	0	0	0	Yes	Yes	Yes	Yes
11	T_23	Tokai	37	Female	ALL	Unrelated	BMT	No	MAC	Zero	85	Dead	0	0	0	Yes	Yes	Yes	Yes
12	T_24	Tokai	53	Female	ALL	Unrelated	BMT	No	MAC	Zero	3685	Alive	0	0	0	Yes	Yes	Yes	Yes
13	T_25	Tokai	34	Male	ALL	Unrelated	CBSCT	No	MAC	Zero	1188	Alive	0	0	0	Yes	Yes	Yes	Yes
14	O_6	Osaka	44	Male	MDS	Unrelated	BMT	No	MAC	Three	304	Dead	2	3	3	Yes	No	No	Yes
15	O_15	Osaka	50	Female	LBL	Related	PBSCT	No	MAC	Three	121	Dead	3	2	0	Yes	No	No	Yes
16	O_24	Osaka	33	Male	AML	Unrelated	BMT	No	MAC	One	1369	Alive	2	0	0	Yes	No	No	No
17	O_30	Osaka	59	Male	AML	Unrelated	BMT	No	RIC	Two	1321	Alive	3	0	0	Yes	No	No	Yes
18	O_31	Osaka	26	Male	AML	Unrelated	BMT	No	RIC	One	1309	Alive	1	0	0	Yes	Yes	No	Yes
19	O_35	Osaka	41	Female	ALL	Unrelated	BMT	Yes	MAC	One	1280	Alive	1	0	0	Yes	No	No	Yes
20	O_39	Osaka	59	Female	ALL	Unrelated	BMT	No	MAC	Two	1181	Alive	3	0	0	Yes	No	No	Yes
22	O_46	Osaka	56	Male	MDS	Unrelated	BMT	No	RIC	Two	1162	Alive	0	1	0	Yes	Yes	No	No
29	T_17	Tokai	20	Male	ALL	Related	BMT	No	MAC	Four	67	Dead	2	4	4	Yes	Yes	Yes	Yes
23	O_49	Osaka	64	Male	AML	Unrelated	BMT	No	RIC	Two	1043	Alive	3	0	0	Yes	No	No	Yes
32	T_26	Tokai	30	Female	Other	Related	BMT	No	MAC	Three	1135	Alive	0	3	0	Yes	Yes	Yes	Yes
33	T_27	Tokai	36	Male	MDS	Unrelated	BMT	No	MAC	Three	1114	Alive	2	3	0	Yes	Yes	Yes	Yes
37	T_31	Tokai	51	Male	CML	Unrelated	BMT	No	MAC	Four	106	Dead	4	2	3	Yes	Yes	No	Yes
21	O_44	Osaka	38	Male	MDS	Unrelated	BMT	Yes	MAC	Three	1201	Alive	2	2	0	Yes	No	No	Yes
28	O_70	Osaka	24	Male	Other	Related	BMT	Yes	RIC	One	727	Alive	1	0	0	Yes	No	No	Yes
24	O_50	Osaka	62	Male	AML	Unrelated	BMT	Yes	MAC	Two	311	Dead	3	1	0	Yes	No	No	Yes
30	T_19	Tokai	58	Male	AML	Related	BMT	No	RIC	One	1289	Alive	2	0	0	Yes	Yes	Yes	Yes
31	T_20	Tokai	31	Female	AML	Related	BMT	No	MAC	Two	1254	Alive	3	0	0	Yes	Yes	Yes	Yes
25	O_58	Osaka	45	Male	MPD	Unrelated	BMT	Yes	MAC	Three	901	Alive	0	4	0	Yes	No	No	Yes
26	O_63	Osaka	56	Female	AML	Unrelated	CBSCT	Yes	RIC	One	792	Alive	0	1	0	Yes	No	No	No
34	T_28	Tokai	48	Female	ALL	Related	BMT	No	MAC	Two	703	Alive	3	0	0	Yes	Yes	Yes	Yes
35	T_29	Tokai	49	Male	ALL	Unrelated	BMT	No	MAC	One	1066	Alive	1	0	0	Yes	Yes	Yes	Yes
36	T_30	Tokai	25	Female	AML	Unrelated	BMT	No	MAC	One	1030	Alive	1	0	0	Yes	Yes	No	Yes
27	O_64	Osaka	44	Male	AML	Related	PBSCT	Yes	RIC	Three	661	Alive	1	3	0	Yes	No	No	Yes
